# Wogonin Strengthens the Therapeutic Effects of Mesenchymal Stem Cells in DSS-Induced Colitis via Promoting IL-10 Production

**DOI:** 10.1155/2021/5527935

**Published:** 2021-06-17

**Authors:** Qiongli Wu, Shujuan Xie, Yinhong Zhu, Jingrou Chen, Jiatong Tian, Shiqiu Xiong, Changyou Wu, Yujin Ye, Yanwen Peng

**Affiliations:** ^1^The Biotherapy Center, The Third Affiliated Hospital, Sun Yat-sen University, Guangzhou 510630, China; ^2^Department of Immunology, Zhongshan School of Medicine, Sun Yat-sen University, Guangzhou 510080, China; ^3^Department of Physics, King's College London, London, UK WC2R 2LS; ^4^Cell Biology Group, National Measurement Lab, LGC, Fordham, Cambridgeshire, UK CB7 5WW; ^5^Department of Rheumatology, The First Affiliated Hospital, Sun Yat-sen University, Guangzhou 510080, China

## Abstract

Inflammatory bowel diseases (IBD) are prevalent and debilitating diseases; their clinical remedy is desperately unmet. Mesenchymal stem cells (MSCs) are pluripotent stem cells with multiple immunomodulatory effects, which are attributed to their efficacy in the IBD rodent model. Optimization of MSC regimes in IBD is a crucial step for their further clinical application. Wogonin is a flavonoid-like compound, which showed extensive immunomodulatory and adjuvant effects. This research is aimed at investigating whether and how Wogonin boosted the therapeutic efficiency of MSCs on DSS-induced colitis. Our results showed that the MSC treatment with Wogonin significantly alleviated the intestinal inflammation in IBD mice by increased IL-10 expression. In vitro experiments, Wogonin obviously raised the IL-10 production and ROS levels of MSCs in a dose-dependent manner. Meanwhile, western blot data suggested Wogonin improves the IL-10 production by inducing transcript factor HIF-1*α* expression via AKT/GSK3*β* signal pathway. Finally, the favorable effects of Wogonin on MSCs were confirmed by IL-10 blockade experiment in vivo. Together, our results suggested that Wogonin significantly increased the IL-10 production and enhanced the therapeutic effects of MSCs in DSS-induced colitis. This work suggested Wogonin as a novel optimal strategy for MSC clinical application.

## 1. Introduction

Inflammatory bowel disease (IBD) is a chronic, relapsing-remitting, inflammatory gastrointestinal disease with a rising incidence worldwide in the past two decades [1]. However, current therapeutic options are far from satisfactory. Recently, cell therapies have been explored in IBD, including mesenchymal stem cells (MSCs) [2]. MSCs are pluripotent stem cells, possessing self-renew ability and multidifferentiation potential function [3]. MSCs showed therapeutic features including angiogenesis, tissue repair, and immunomodulation, among which IL-10 was an important effector molecule [4]. Several researches supported that IL-10 is highly relevant to IBD, and IL-10^−/−^ mice would spontaneously develop colitis [5]. In humans, polymorphisms in IL-10 [6] have been found to be correlated with very early onset of colitis. Our previous studies on rodent models of IBD also showed that MSC administration could significantly improve intestinal inflammation via IL-10 [7, 8]. Consistently, some strategies have been proposed to improve the therapeutic efficiency of MSC, and engineering IL-10 overexpressing MSCs have achieved better therapeutic efficacy in immune relative diseases [9–11].

Accumulating researches indicated the extracts of herbal plants have diverse biological activities [12]. *Scutellaria baicalensis* is widely used as Chinese herbal medicine, and Wogonin is one of its major bioactive compounds [13]. Wogonin is a flavonoid-like compound which possesses anticancer and immunomodulatory effects [14]. Although there is evidence that both MSCs and Wogonin could alleviate IBD [15–20], it is still unclear whether combining MSCs with Wogonin be more superior to either therapy alone.

In this research, we demonstrated that Wogonin extended the therapeutic efficiency of MSCs on DSS-induced murine colitis in vivo. It was due to the increased IL-10 expression in the intestinal tissue and peritoneal cavity. Mechanistic studies suggested that Wogonin enhanced IL-10 production and ROS levels in MSCs via AKT/GSK3*β* signal pathway. Furthermore, in vivo, the data exhibited that neutralizing anti-IL-10 antibody can abrogate the therapeutic effects of a combination of MSCs and Wogonin on colitis.

## 2. Material and Methods

### 2.1. Animals

Male C57BL/6 mice aged 8-12 weeks were purchased from the Nanjing Model Experimental Animal Center and housed in pathogen-free conditions at Sun Yat-sen University. The age- and weight-matched of mice were applied for all mouse-related experiments. All animal studies were approved by the Zhongshan School of Experimental Animal Ethics Committee, Sun Yat-sen University, Guangzhou, China, and in strict compliance with the corresponding guidelines.

### 2.2. Reagents and Antibodies

Reactive Oxygen Species Detection Assay Kit was purchased from BioVision (San Francisco, CA, USA). Wogonin was purchased from MedChemExpress (Israel Shekel), and LPS was purchased from Sigma-Aldrich (St. Louis, MO, USA). The following antibodies were used for cell surface staining: CD34-PE, CD44-FITC, CD29-PE-Cy7, Sca-1-AF700, CD45-AF700, and CD49e-APC are all from BioLegend and Mouse IL-10 ELISA Set and Zombie Green™ Fixable Viability Kit were purchased from BD Biosciences. Mouse IL-10 MAb was purchased from R&D Systems.

### 2.3. Induction and Assessment of Colitis

Dextran sulfate sodium salt (DSS) was purchased from MP Biomedicals (United States) which was used to induce acute colitis in mice. 3% (*w*/*v*) DSS was dissolved in sterile water and supplied to the mice in drink water for 7 days, followed by regular drinking water until day 8. Mice were randomly divided into five groups, and there were 6 mice in each group: the mice were fed regular water as the control group, were exposed to 3% DSS water as the IBD group, were administrated 1 × 10^6^ MSCs via peritoneal injection as the MSC group, and were injected 10 mg/kg Wogonin via i.p. as the Wogonin group, and the combination of MSC and Wogonin group received both 1 × 10^6^ MSCs and 10 mg/kg Wogonin. Meanwhile, body weights of animals were scaled and recorded daily. Eight days later, the animals were sacrificed, and the length of the colon from the cecocolic junction to the anal verge was measured. Severity of the disease was assessed by weight loss, colon length, and histopathology scores of the colon. Colon tissues were processed for histological analysis by hematoxylin and eosin (H&E) stain. Histology was used to evaluate the inflammation (I) (0, none; 1, slight; 2, moderate; 3, severe), extent (E) (0, none; 1, mucosa; 2, mucosa and submucosa; 3, transmural), regeneration (R) (4, no tissue repair; 3, surface epithelium not intact; 2, regeneration with crypt's depletion; 1, almost complete regeneration; 0, complete regeneration or normal tissue), crypt damage (C) (0, none; 1, basal 1/3 damaged; 2, basal 2/3 damaged; 3, only surface epithelium intact; 4, entire crypt and epithelium lost), and percentage involvement (P) (1, 1%–25%; 2, 26%–50%; 3, 51%–75%; 4, 76%–100%) according to previous reports [21]. The division animal groups and the histological scoring system are listed in Table 1.

### 2.4. HE Staining

Colon tissue was removed and then immersed in 4% paraformaldehyde. After dewaxing with dehydrated alcohol gradient, the tissue was embedded in paraffin and stained with hematoxylin and eosin (H&E).

### 2.5. MSC Differentiation Assays

MSCs were isolated from the bone marrow of 6-8 weeks C57BL/6 mice, according to the improved low-density culture method [22]. The StemPro™ Osteogenesis Differentiation Kit (Thermo Fisher Scientific) and StemPro™ Adipogenesis Differentiation Kit (Thermo Fisher Scientific) were used for osteogenic and adipogenic differentiation in vitro, according to the manufacturer's recommended procedures. All cells at 6-8 passage were used in this study.

### 2.6. ELISA

Supernatants of cultured cells and mouse peritoneal lavage fluid samples were collected for detection of mouse IL-10 by enzyme-linked immunosorbent assay (ELISA Kit IL-10 from BD Biosciences, San Jose, CA, USA). The sensitivities of ELISA kits were 31.2 pg/ml for IL-10. ELISA assays were performed according to the manufacturer's instructions.

### 2.7. RNA-seq

After 24 h Wogonin treatment, RNA was extracted from both untreated MSCs and treated MSCs using Trizol (Invitrogen, Carlsbad, CA). RNA-seq data were normalized through the DESeq2 pipeline, and gene heat map was fulfilled with packages pheatmap under R 4.0.2. The gene ontology analysis was performed based on clusterProfiler written by Yu et al. [23].

### 2.8. Western Blot

Cells or homogenized tissue were lysed in ice-cold RIPA buffer (50 mM Tris-HCl pH 8.0, 150 mM sodium chloride, 1% NP-40, 0.5% sodium deoxycholate, 0.1% sodium dodecyl sulfate, 2 mM EDTA containing 1x protease inhibitor cocktail, and phosphatase inhibitor (Roche)). Equivalent total protein extracts were separated by SDS-polyacrylamide gel electrophoresis (PAGE) and transferred to nitrocellulose membrane (GE Healthcare Life Sciences, Germany). After that, membranes were blocked with 5% bovine serum albumin (BSA) for 1 h at room temperature. Primary antibodies were incubated, and a horseradish peroxidase-conjugated secondary antibody was followed. The primary antibodies used were as follows: antibodies for TNF-*α* antibodies (#11948T), HIF-1*α* antibodies (#4914S), p-GSK3*β* (#9323T), GSK3*β* (#9315S), p-AKT (#4060S), AKT (#4691S), p-Stat3 (#9145S), Stat3 (#9404S), p-JNK (#4671), JNK (#9258), and GAPDH (#2118) were obtained from Cell Signaling Technology; all those antibodies were diluted with antibody solution and used at a dilution of 1 : 2000. IL-10 (sc-365858) was obtained from Santa Cruz and employed in a 1 : 500 dilution. The secondary antibodies were used at dilution 1 : 5000. All proteins were visualized by Chemiluminescent HRP Substrate (Millipore, WBKLS0500), and chemical luminescence of membranes was detected by the Bio-Rad luminescent imaging system. The gray value of each protein bands was quantified by Image J software.

### 2.9. Flow Cytometry

For cell surface marker staining, the cells were washed with PBS buffer containing 0.1% BSA and 0.05% sodium azide and labeled with surface markers and dead/live streaming antibodies for 30 min at 4°C in dark, then washed twice with PBS buffer containing 0.1% BSA and 0.05% sodium azide. All the above stained cells were assayed by FACS Aria II (Becton Dickinson, San Jose, USA), and the data were analyzed by FlowJo software (TreeStar, San Carlos, USA).

For ROS detection, CS&T beads (BD, 661414) were used for machine calibration and performance tracking. BD rainbow beads were for PMT voltage optimization (BD, 559123). MSCs were treated with Wogonin (0, 12.5, 25, and 50 *μ*M) in the presence of LPS. After 24 h, cells were incubated with 20 *μ*M DCF-DA at 37°C for 20 min, then detected by flow cytometry. Fluorescence minus one (FMO) was applied for positive cell gating. Anti-rat Igk negative control compensation particle set (BD, 552845) was used for fluorochrome compensation.

### 2.10. IL-10 Blockade Experiment In Vivo

Eight to twelve weeks C57BL/6 male mice were exposed to 3% DSS water for 7 days, then fed the regular water. On the 2nd day, all mice were peritoneal injected 1 × 10^6^ MSCs and 10 mg/kg Wogonin. Three of them additionally received neutralizing anti-mouse IL-10 antibody (R&D Systems) at a dose of 100 *μ*g/mouse via peritoneal injection. On day 8, the colonic length and pathological lesions of the colon were detected.

### 2.11. Statistical Analysis

All statistical tests were performed with GraphPad Prism 7 (GraphPad Software Inc., San Diego, USA). Significant differences between data sets were performed with either the unpaired Student's *t* test when comparing two groups, one-way ANOVA for more than two groups, or two-way ANOVA for two variables (GraphPad Software Inc., San Diego, CA, USA). Data were represented as mean or mean ± SEM. ^∗∗∗∗^*P* < 0.0001; ^∗∗∗^*P* < 0.001; ^∗∗^*P* < 0.01; ^∗^*P* < 0.05; and *P* > 0.05, not significant, as stated in figure legends.

## 3. Results

### 3.1. Wogonin Enhanced the Therapeutic Effects of MSCs on DSS-Induced Colitis via Increasing IL-10

**Figure 1:**
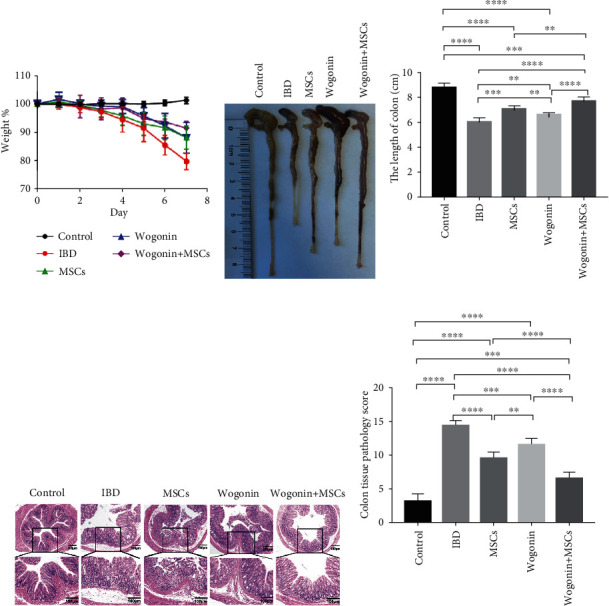
A combination of MSCs and Wogonin possessed optimal therapeutic effects on DSS-induced colitis. Mice were administered 3% DSS in drinking water for 7 days, followed by regular drinking water. On the 2nd day, mice received MSC treatment, Wogonin treatment, or a combined treatment, respectively. Then, mice were euthanized on day 8. (a) Body weights were monitored daily, and the values were expressed as percentage weight loss. (b, c) Colon length of five groups of mice was measured and recorded on day 8. (d) Representative H&E staining of the distal colon. Scale bar = 100 micros. Magnification, 40x and 100x. (e) Colonic histopathological scores of all mice were evaluated on day 8. Data represent mean values ± SEM. The statistical significance of difference between two means was calculated with an unpaired. A one-way ANOVA was performed, ^∗∗^*P* < 0.01; ^∗∗∗^*P* < 0.001; ^∗∗∗∗^*P* < 0.0001. *n* = 6/group.

To explore the influence of Wogonin on the therapeutic effects of MSCs, we compared the effect of MSCs or Wogonin alone, and the effect when combined. From day 5, weight loss was observed in all mice except the control mice. As shown in Figure 1(a), the IBD mice lost most weight, but the combination of MSC and Wogonin group had the least weight loss. In this study, colon length was also recorded as an index of mouse colitis. On the 8th day, all colon tissues were collected and measured. The results indicated that the length of colon tissues from all mice exposed to DSS was shorter than those from the control groups. Interestingly, the colons from the combination group were significantly longer than those from the Wogonin or MSC groups (Figures 1(b) and 1(c)). This implied that a combination of Wogonin and MSCs possessed better therapeutic effects on murine colitis than using them separately. Meanwhile, colon histopathological examination consistently showed less intestinal inflammation in the combination group comparing to the other treatment groups (Figure 1(d)). The pathological score of the combination of MSCs and Wogonin-treated mice was the lowest in all treated mice (Figure 1(e)).

**Figure 2:**
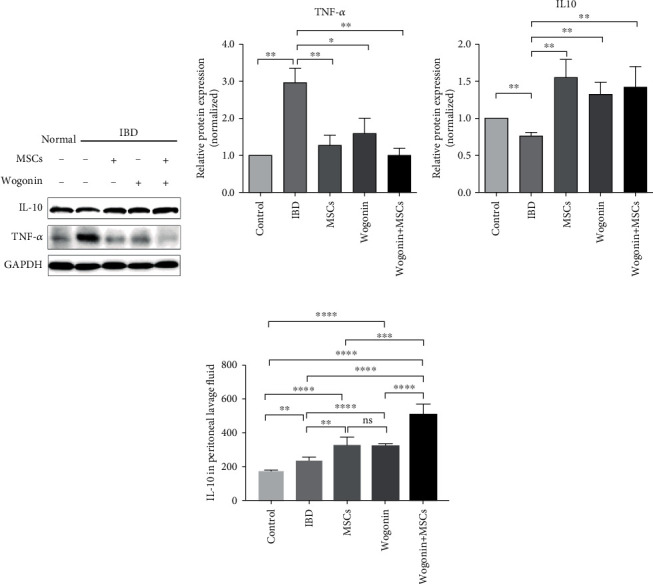
MSCs and Wogonin increased IL-10 levels in both colon tissue and peritoneal lavage fluid. Mice were administered 3% DSS in drinking water for 7 days, followed by regular drinking water. On the 2nd day, mice received MSCs, Wogonin, or a combined treatment, respectively. The colon tissue and peritoneal lavage fluid were collected on 8 d. (a) The expression of TNF-*α* and IL-10 in the colon was detected by western blot, and the results represented three independent experiments. (b) TNF-*α* and (c) IL-10 expressions were calculated separately. (d) The IL-10 levels of peritoneal lavage fluid were measured by ELISA. Data represent mean values ± SEM. The statistical significance of difference between two means was calculated with an unpaired. *n* = 6/group. A one-way ANOVA was performed, ^∗^*P* < 0.05; ^∗∗^*P* < 0.01; ^∗∗∗^*P* < 0.001; ^∗∗∗∗^*P* < 0.0001; ns: no significance.

To investigate the possible therapeutic mechanisms, the expression of TNF-*α* and IL-10 in colon was determined by western blot. The data suggested that DSS administration significantly enhanced the expression of proinflammatory cytokine TNF-*α* and reduced the expression of anti-inflammatory cytokine IL-10 in the colon tissue. No matter whether MSCs and Wogonin are used together or alone, they could significantly reduce local TNF-*α* and increased local IL-10 (Figures 2(a)–2(c)). To further inspect the change of cytokines, the levels of TNF-*α* and IL-10 in peritoneal lavage fluids were detected by ELISA. However, the level of TNF-*α* was too low to be detected. In line with western blot data, IL-10 significantly increased in peritoneal lavage fluids from the combination group comparing to the MSC or Wogonin groups. This result suggests that IL-10 may be the key factor for Wogonin strengthening the therapeutic effect of MSC (Figure 2(d)).

### 3.2. Wogonin Promoted IL-10 Production of MSCs

**Figure 3:**
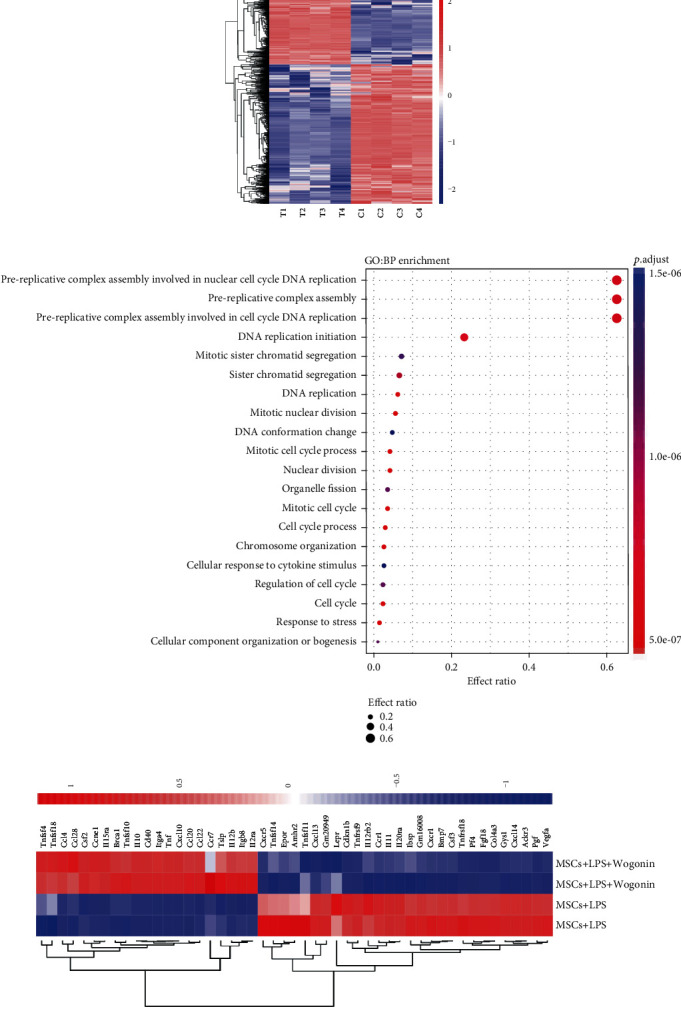
Transcriptome analysis of Wogonin-treated MSCs. (a) The heat map showed the transcriptome changes of Wogonin-treated MSCs and untreated MSCs. (b) Gene ontology analysis on two different groups. (c) Heat map gene expression of cytokine and chemokines in MSCs, each gene expression was scaled based on heat map packages.

To understand how Wogonin strengthened the therapeutic effects of MSCs, we compared the difference of gene expression profiles between untreated MSCs and Wogonin-treated MSCs in the presence of LPS. After 24 h stimulation, untreated MSCs and Wogonin-treated MSCs were collected, and the transcriptome analysis was performed. The transcriptome analysis identified the upregulation of 1000 genes and downregulation of 2000 genes in Wogonin-treated MSCs (Figure 3(a)). GO analysis indicated that the upregulated genes were highly enriched in the cell cycle (Figure 3(b)).

Furthermore, RNA-seq analysis also revealed that Wogonin enhances the expression of various chemokines and IL-10 in MSCs (Figure 3(c)).

**Figure 4:**
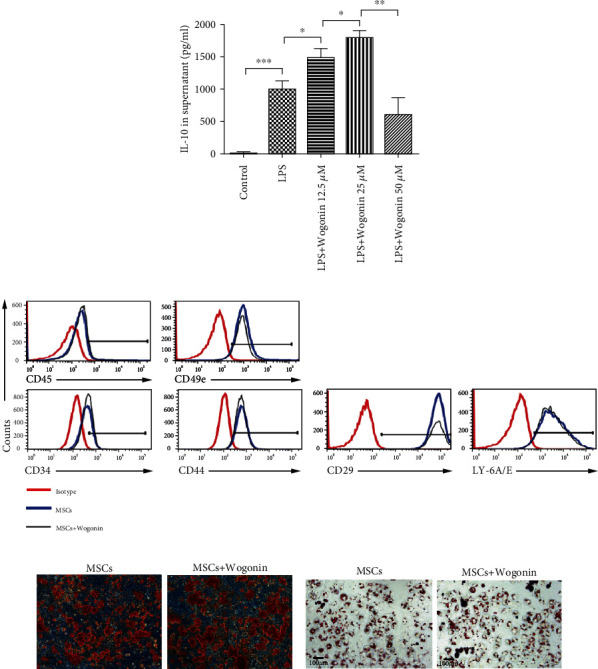
Wogonin increased the IL-10 production of MSCs. (a) MSCs were stimulated by Wogonin at a different dose (12.5, 25, and 50 *μ*M) in the presence of LPS. 24 h later, the IL-10 level in supernatant was detected by ELISA. Data represent mean values ± SEM of three independent experiments. The statistical significance of difference between two means was calculated with an unpaired. A one-way ANOVA was performed, ^∗^*P* < 0.05; ^∗∗^*P* < 0.01; ^∗∗∗^*P* < 0.001. (b) After 24 h stimulation of 25 *μ*M Wogonin, the surface markers of both untreated MSCs and Wogonin-treated MSCs were analyzed by flow cytometry. (c) The osteogenic differentiation was showed by Alizarin red S staining. (d) The adipogenic differentiation was shown by Oil O staining.

To further verify the influence of Wogonin on the IL-10 production of MSCs, we activated MSCs with different concentrations of Wogonin in the presence of LPS in vitro. After 24 h stimulation, the supernatant was collected, and the IL-10 level was detected by ELISA. As shown in Figure 4(a), the IL-10 secretion from MSCs gradually increased in a dose-dependent manner, reached the peak at 25 *μ*M, then decreased when continuously increasing Wogonin dosage. It was illustrated that 25 *μ*M was the optimal dose of Wogonin to increase the IL-10 production in MSCs.

To clarify whether Wogonin affects the characteristics of MSCs, we firstly analyzed surface markers of untreated MSCs and 25 *μ*M Wogonin-treated MSCs. Comparing to untreated MSCs, MSCs expressed the same panel of surface markers after Wogonin treatment, including CD29, CD44, CD49e, and Sca-1, and did not express the hematopoietic stem cell markers CD34 or CD45 (Figure 4(b)). Additionally, osteogenic differentiation and adipogenic differentiation were tested. As confirmed by Alizarin red S staining and Oil O staining, respectively, Wogonin-treated MSCs showed no change in mesodermal differentiation capacity compared with untreated MSCs (Figures 4(c) and 4(d)).

### 3.3. Wogonin Increased ROS Level in MSCs

**Figure 5:**
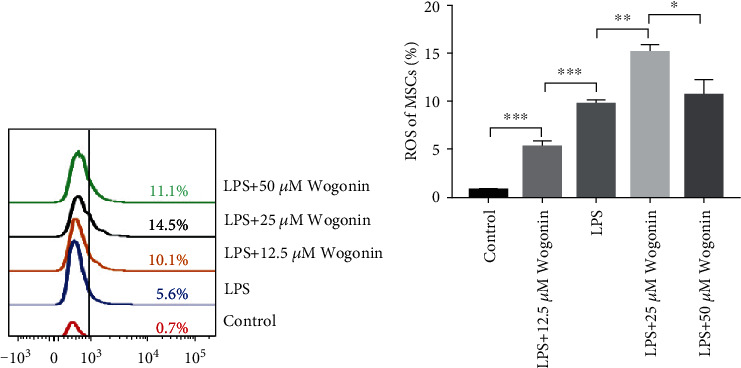
Wogonin upregulated ROS level of MSCs. MSCs were stimulated by Wogonin at a different dose (12.5, 25, and 50 *μ*M) in the presence of LPS. 24 h later, the ROS level in MSCs was detected by flow cytometry. (a) Representative histogram of ROS levels of MSCs. (b) The results represented three independent experiments for quantification of ROS levels in MSCs. Values are means ± SEM. The statistical significance of difference between two means was calculated with an unpaired. A one-way ANOVA was performed, ^∗^*P* < 0.05; ^∗∗^*P* < 0.01; ^∗∗∗^*P* < 0.001.

To investigate the effects of Wogonin on ROS level in MSCs, different doses of Wogonin were used to stimulate MSCs in vitro, and the ROS levels of MSCs were detected by DCFDA staining and analyzed by flow cytometry. The results showed that Wogonin treatment obviously increased the ROS level of MSCs in a dose-dependent manner, being greatest at 25 *μ*M (Figures 5(a) and 5(b)). When Wogonin dose was increased to 50 *μ*M, ROS level decreased instead, which was consistent with the IL-10 expression of MSCs (Figures 3(a) and 3(b)). These data implied that Wogonin treatment regulated the ROS level of MSCs.

### 3.4. Effect of Wogonin on the GSK3*β*/AKT Signaling Pathway of MSCs

**Figure 6:**
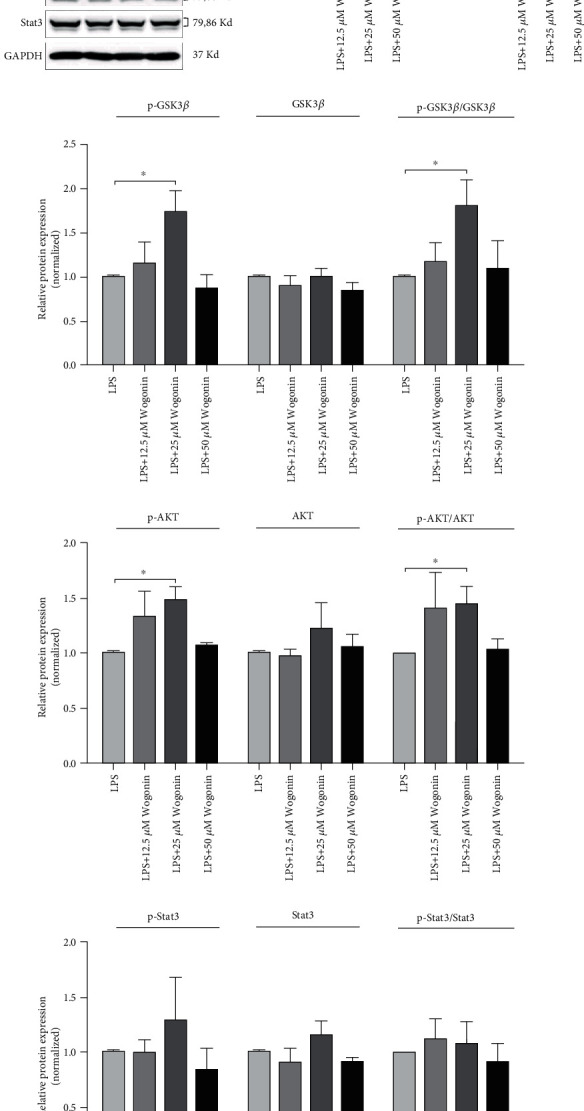
Wogonin improves the IL-10 production in MSCs via GSK3*β*/AKT pathway. MSCs were stimulated by Wogonin at a different dose (12.5, 25, and 50 *μ*M) in the presence of LPS. After 24 h stimulation, western blot was performed. The cell extract probed with antibodies as indicated, and GAPDH as loading control. Relative protein expression was calculated, respectively. (a) The representatives of the protein expression of HIF-1*α*, IL-10 as well as GSK3*β*, AKT, and Stat3 and their phosphorylated forms (p-GSK3*β*, p-AKT, and p-Stat3). The effects of Wogonin on the expression of HIF-1*α* and IL-10. (b) The effects of Wogonin on GSK3*β*. (c) AKT (d) and Stat3 (e) signaling pathways. All the western blotting experiments were repeated three times. Values are means ± SEM. The statistical significance of difference between two means was calculated with two-way ANOVA followed by multiple comparisons with *t* test and Bonferroni correction, ^∗^*P* < 0.05.

To elucidate the potential signaling pathways of Wogonin increasing IL-10 production in MSCs after Wogonin treatment, we tested protein expression level IL-10 of MSCs that were cultured with LPS in the absence or presence of Wogonin at different dose firstly. A role for transcript factor HIF-1*α* involved in IL-10 expression was well established; therefore, the expression of HIF-1*α* was also detected. Consistent with IL-10 in MSC supernatant, we found that HIF-1*α* and IL-10 gently upregulated in the presence of Wogonin treatment, significantly on 25 *μ*M dose, but decreased on 50 *μ*M dose (Figures 6(a) and 6(b)). Next, signaling pathways involved in IL-10 production were assessed, and western blot analysis showed a remarkable increase of phosphorylation of glycogen synthase kinase 3*β* (GSK3*β*) and phosphorylation of AKT in the presence of Wogonin with 25 *μ*M, while total GSK3*β* and AKT changed slightly (Figures 6(a), 6(c), and 6(d)). Phosphorylation of Stat3 signaling kept intact on Wogonin treatment (Figures 6(a) and 6(e)). These results suggested that Wogonin upregulates the IL-10 production involved in GSK3*β*/AKT signaling pathways.

### 3.5. IL-10 Blockade Abrogated the Therapeutic Effects of a Combination of MSCs and Wogonin on Colitis

**Figure 7:**
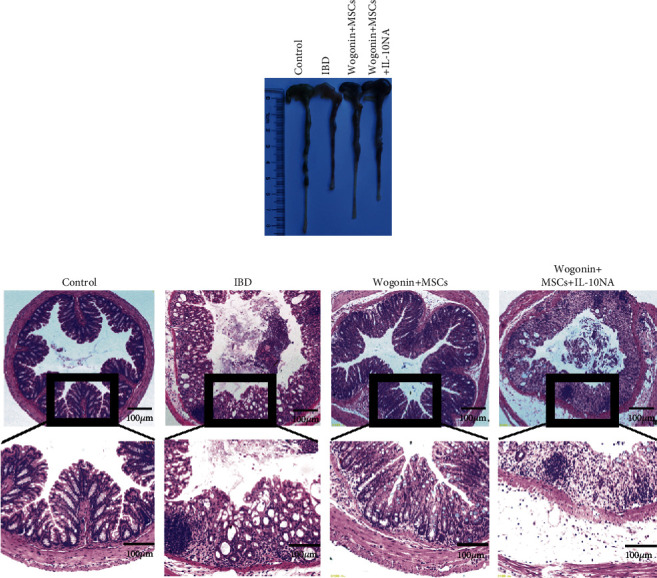
IL-10 blockade abrogated the therapeutic effects of a combination of MSCs and Wogonin on colitis. On day 2 after exposing to 3.0% DSS water, mice received a combination of MSCs and Wogonin or additionally add neutralizing anti-IL-10 antibody, respectively. The colon length and histological analysis were performed on day 8. (a) Representative of colon length from the different groups. (b) Representative images of H&E staining slides. Magnification, 40x and 100x. Three independent experiments showed similar results.

To confirm the crucial role of IL-10 on ameliorating mouse colitis, neutralizing IL-10 antibody was used for the combination of MSCs and Wogonin mice. As shown in Figure 7, IL-10 blockade abolished the improvement of the combination on murine intestinal inflammation. After IL-10 neutralization, the shorter colon length was observed, compared with the no neutralization mice (Figure 7(a)). HE staining data also suggested that more serious intestinal epithelial structure damage and more inflammatory cell infiltration were observed in the colon tissues from the IL-10 neutralization mice than those from no IL-10 neutralization mice (Figure 7(b)). These results further demonstrated that the increased production of IL-10 in MSCs is a key factor for Wogonin to improve the therapeutic efficacy of MSC on DSS-induced colitis.

## 4. Discussion

Ulcerative colitis (UC), along with Crohn's disease (CD), is chronic and debilitating inflammatory bowel disease (IBD). IBD has been reported to have high prevalence with increasing trends in the rate of incidence [24]. The hygiene hypothesis is the most popular explanation for this growing incidence; meanwhile, the particular etiological factors were still enigma [25]. The impairment of colon mucus layer barrier and epithelium and change of luminal microbial diversity (dysbiosis) were commonly observed in the pathogenesis of UC. TNF-*α* and multitude of Th2-derived cytokines were reported to be involved in this process [26]. The lack of a cure or effective long-term treatment options has resulted in substantial morbidity of IBD [27], which highlighted the urgent demand of investigation of a new therapeutic regime.

Mesenchymal stem cells (MSCs) are multipotent stromal cells which can differentiate into different cell types according to their residential niches. The cells sprinted into the clinic stage center due to their features of versatile development potential, self-renewal, allogenic compatibility, and immunomodulatory effects. MSCs are currently being widely tested as a cellular therapy in chronic intestinal diseases, including IBD, and showed promising clinical outcomes with unmet optimization and improvement [28–30], such as in vivo low level of MSC recruitment, and persistence decreased their therapeutic efficacy [31].

To overcome these hurdles, IFN-*γ*, TGF-*β*, IGF-1C, IL-1*β*, and PGE2 were applied to precondition MSCs before they were administered onto different experimental models, and pretreated MSCs showed an enhanced therapeutic effect, with different limitations [32–36]. All of these promising explorations encouraged us to search further alternatives.

Wogonin is a natural flavonoid component extracted from the dried root of *Scutellaria baicalensis*. Various studies showed its direct anti-inflammatory effects and immunoregulation on immune cells [37]. Interestingly, Wogonin was proved as a particular therapeutic adjuvant for anti-inflammation [14], which inspired our explorations of the potential role of Wogonin in MSC medication on IBD.

On our solid and highly repeatable DSS-induced mouse colitis model, MSCs and Wogonin showed an additive or synergetic effect on the mitigation of colitis. As expected, TNF-*α* amount in inflammatory colon tissue was decreased by MSCs, Wogonin alone, and their combinations. This was consistent with the alleviated intestinal inflammation by pathology score, which echoed by previous reports.

As another key player of inflammation, IL-10 is an anti-inflammatory factor, playing a critical role in the suppression of autoimmune reactivity and in termination of the inflammatory response. IL-10 from T cell have been established an anti-inflammatory effect in IBD [38], and IL-10 secreted from MSCs have been proved to attenuate inflammation in mice [9–11, 39, 40]. Our recent report showed Wogonin regulated IL-10 production [41]. Hence, it is interesting to study the effect of Wogonin on IL-10 production from MSC in our mouse model.

Strikingly, IL-10 amount in peritoneal lavage fluid was increased by Wogonin and MSCs additively, which did not show in intestinal tissues. Comparably, TNF-*α* amount in peritoneum was under detectable range. This indicated that Wogonin might directly alter peritoneal MSCs on-site.

To verify this hypothesis and examine the full profile of the effect of Wogonin on MSCs from a different level, LPS-activated MSCs cocultured with/without Wogonin in vitro.

Firstly, Wogonin enhanced abundant proliferation-associated gene expression in MSCs, but not those development-associated genes. This was consistent with no surface, and differentiation markers were changed by Wogonin culture. Meanwhile, both TNF-*α* and IL-10 mRNA amounts were augmented along with the activation of numerous chemokine genes. This echoed with the increased protein amount of peritoneal IL-10.

Secondly, there is a clear dose-effect of Wogonin enhancing IL-10 production from MSCs. 25 *μ*M Wogonin showed optimal augment effect, and higher concentration suppressed further IL-10 secretion. Reactive oxygen species (ROS) generation by NADPH oxidase has been shown to interact with IL-10 production in macrophages [42, 43]. Hence, it is not surprising to repeat this on MSCs. All these results indicated the adjuvant feature of Wogonin on MSC activation.

To further investigate the molecular machinery of Wogonin on MSCs, TLR4 signaling pathways were explored. Strikingly, the TLR4/AKT/GSK3*β* pathway was triggered by Wogonin. It is still difficult to pinpoint the target receptor on MSCs due to the herb derivative, and their discovery of this machinery on MSCs suggested the new strategy in IBD therapy [44]. Our in vivo IL-10 neutralization data reinforced this concept.

To our knowledge, our study firstly showed Wogonin enhanced IL-10 production from MSCs directly, which restrained DSS-induced colitis. This implied new therapeutic strategy for IBD.

## 5. Conclusions

Although MSCs show promising therapeutic potential in treating inflammatory bowel disease (IBD), their efficacy is influenced by multiple factors. Wogonin, a bioactive compound from *Scutellaria baicalensis*, has diverse immunoregulatory effects. Our present study demonstrated that Wogonin extended the therapeutic efficiency of MSCs on DSS-induced murine colitis in vivo, which was due to the increased IL-10 expression in the intestinal tissue and peritoneal cavity. RNA sequencing analysis suggested Wogonin enhanced IL-10 expression, which was confirmed via in vitro experiments. Furthermore, western blot data implied that Wogonin upregulates transcript factor HIF-1*α* in MSCs via AKT/GSK3*β* signal pathway. Summarily, our study found that combining MSCs with Wogonin was superior to MSC therapy alone, which provides a novel strategy for MSC clinical application on IBD.

## Figures and Tables

**Table 1:** The division animal groups and the histological scoring system.

Animal groups	Tissue inflammation	Extent	Regeneration	Crypt damage	Percentage involvement	Histological score
Control/	None	None	Complete regeneration or normal tissue	None	—	0
IBD/	Slight	Mucosa	Almost complete regeneration	Basal 1/3 damaged	1%–25%	1
MSCs/	Moderate	Mucosa and submucosa	Regeneration with crypt's depletion	Basal 2/3 damaged	26%–50%	2
Wogonin/	Severe	Transmural	Surface epithelium not intact	Only surface epithelium intact	51%–75%	3
Wogonin+MSCs	—	—	No tissue repair	Entire crypt and epithelium lost	76%–100%	4

## Data Availability

The data used to support the findings of this study are included within the article.
